# How risky are second trimester clandestine abortions in Cameroon: a retrospective descriptive study

**DOI:** 10.1186/1472-6874-14-108

**Published:** 2014-09-09

**Authors:** Elie Nkwabong, Robinson Enow Mbu, Joseph Nelson Fomulu

**Affiliations:** 1 Department of Obstetrics & Gynecology, University Teaching Hospital & Faculty of Medicine and Biomedical Sciences, PO Box 1364, Yaoundé, Cameroon; 2 Department of Obstetrics & Gynecology, Central Maternity & Faculty of Medicine and Biomedical Sciences, Yaoundé, Cameroon

**Keywords:** Clandestine second trimester abortions, Clandestine first trimester abortions, Complications

## Abstract

**Background:**

Complications of clandestine abortions increase with gestational age. The aim of this study was to identify complications of second trimester clandestine abortions (STA) and those of first trimester clandestine abortions (FTA).

**Methods:**

This retrospective descriptive study was conducted between March 1st and August 31st, 2012 in the University Teaching Hospital and the Central Hospital, Yaoundé (Cameroon). The files of women with clandestine abortions carried out outside our units, but received in our settings for some complications were reviewed. Variables studied were maternal age, parity, marital status, gestational age at the time of abortion, the abortion provider and the method used, the duration of antibiotic coverage, the time interval between abortion and consultation, the complications presented and the duration of hospital stay. Data of 20 women with STA (≥13 weeks 1 day) and those of 74 women with FTA (≤13 complete weeks) were analyzed and compared. The t-test was used to compare continuous variables. P value <0.05 was considered statistically significant.

**Results:**

Women with STA had high parities (P = 0.0011). STAs were mostly performed by nurses and were usually done by dilatation and curettage or dilatation and evacuation, manual vacuum aspiration, intramuscular injection of an unspecified medication, transcervical foreign body insertion, amniotomy and misoprostol. STA complications were severe anemia, hypovolemic shock, uterine perforation and maternal death.

**Conclusions:**

Clandestine abortions, especially second trimester abortions, are associated with risks of maternal morbidity and mortality especially when done by nurses. Therefore, women should seek for help directly from trained health personnel (Gynecologists & Obstetricians). Moreover, nurses should be trained in uterine evacuation procedures. They should also refer women who want to carry out STA to Gynecologists and Obstetricians. Finally, to reduce the prevalence of abortion in general, the government should make contraception available to all women, as well as use public media to sensitize women on the dangers of abortion and on the need to use family planning services.

## Background

The rate of unwanted pregnancies is still increasing, despite availability of contraceptive methods, due to the rise in number of women of reproductive age, non- use, incorrect use or failure of contraceptive methods [1]. Unwanted pregnancies are associated with high rates of induced abortions [2]. Induced abortions, whether clandestine or not, carry more risks of complications than spontaneous abortions. These risks increase with gestational age. First trimester abortions carry lesser risks of complications than second trimester abortions [3,4]. It is estimated that between 47,000 and 66,500 women worldwide die yearly as a consequence of unsafe abortions [1,5] while a very larger number are cases of near-miss with short and long term health sequelae [5].

Abortion is illegal in Cameroon [6,7], explaining why some unwanted pregnancies end in clandestine abortions. Abortion is legal in Cameroon only in cases of rape or incest [6,8]. In our country, some women have limited knowledge on sexual education, contraception and reproductive health and only few women (14%) use modern contraceptive methods [9]. Moreover, some of these women do not know early signs of pregnancy. Consequently, some of them realize that they are pregnant usually in the second trimester and sometimes with quickening. At this moment some of these women still choose to terminate the pregnancy -although in the second trimester- as already noticed by some authors [10,11].

Unsafe abortion is 8 to 10 times riskier than childbirth [12,13], but when abortion is done in safe conditions, it is 14 times less risky than childbirth [14]. Cases of first trimester clandestine abortions (FTA) and occasionally second trimester clandestine abortions (STA) are received in our services each month. Since complications of abortion increase with gestational age, we expect more complications among women with STA. No study on second trimester clandestine abortions has been carried out in our environment. The aims of this study, therefore, were to identify the women receiving services, the abortion methods used, the abortion providers and the complications experienced by women with clandestine abortions, especially STA.

## Methods

This retrospective descriptive study was conducted between March 1st and August 31st, 2012 in the University Teaching Hospital and the Central Hospital, Yaoundé (Cameroon). Files of women with clandestine abortions (done outside our units but who were received later in our settings for some complications) were retrieved from archives and reviewed. Admission registers were used to identify the women (with their file number) who carried out clandestine abortions.

Habitually in our services, women who have undergone clandestine abortions are received in our setting by the resident, who takes the history. If the information given by the patient seems too confidential but important, it is verified later by asking the same question otherwise. Some informations are obtained from the patient’s referral notes, if available. The blood pressure, pulse rate and body temperature are taken and the patient’s physical examination is done by the resident and eventually with the gynecologist. This physical examination emphasizes on the patient’s general status, the conjunctivae (well colored or not), the abdomen and the pelvis (tender or not), the size and consistency of the uterus, the cervix (opened or not) and the characteristics of the vaginal discharge after which an initial diagnosis is made. The investigations are prescribed under the gynecologist’s supervision. These include the full blood count (done on venous blood with HumaCount 30TS), blood group, blood clotting profile (prothrombin time) and pelvic ultrasound scan. After explaining the final diagnosis and the different treatment modalities to the patient, an informed consent is obtained from her. Then, the proposed treatment is done by the gynecologist or under his supervision. This may include: uterine evacuation with manual vacuum aspiration (for incomplete abortion), administration of antibiotics (for treatment of pelvic infection which is characterized by uterine or pelvic tenderness with offensive vaginal discharge), administration of uterotonics, blood transfusion (if severe anemia defined as hemoglobin concentration <7 g/dl is diagnosed), laparotomy if uterine perforation is suspected (pelvic tenderness with or without fever) or if there is generalized peritonitis (fever, generalized abdominal tenderness), or admission in our units for follow-up. This is performed under the gynecologist’s supervision. All data collected from admission to discharge are written in patients’ files.

In our study, we wanted to know the socio-demographic characteristics of women who underwent clandestine abortions, the conditions in which they were done and the complications presented. The data were extracted from patients’ files by the principal investigator or the senior resident in obstetrics and gynecology working in each of the two hospitals. Data collected on a pre-established questionnaire included maternal age at abortion, parity (number of deliveries after 28 complete weeks gestation), marital status, gestational age at the time of abortion (validated by an ultrasound scan if any, or calculated from the last menstrual period and confirmed by bimanual vaginal examination), the abortion provider (obtained from patient), the place where abortion was done (the name of the heath institution), the method used (obtained from patient’s description of the procedure done), the duration of antibiotic coverage (the number of days of oral antibiotic therapy), the time interval between abortion and consultation in our units, the complications presented and the duration of hospital stay.

The necessary sample size was calculated as needing at least 79 women who presented with complications following clandestine abortions. This research has adhered to the STROBE guidelines for observational studies (Additional file 1). This study received approval from the ethics committees of the University Teaching Hospital and of the Central Hospital, both of Yaoundé (Cameroon). Data were subsequently analyzed using SPSS 18.0. Data of women with second trimester (≥13 weeks 1 day) clandestine abortions and those of women with first trimester (≤13 complete weeks) clandestine abortion were analyzed. The t-test was used to compare continuous variables. The significance level was P < 0.05. Results are presented as mean ± standard deviation (SD) for quantitative data and frequencies for qualitative data.

Footnote: To calculate the sample size, the following formula for descriptive studies N = P(1-P) Zα^2^/D^2^ was used where Zα = 1.96 corresponds to a confidence level of 0.05, D = 0.06 is the degree of precision and assuming that the prevalence of complications following clandestine abortion (P) might be 8% in Yaoundé.

## Results

Ninety four clandestine abortions were recorded during the study period amongst which 74 first trimester abortions and 20 second trimester abortions. Some data are summarized in Table 1.

**Table 1:** Distribution of some variables among both groups

**Variables**	**FTA**	**STA**	**P value**
Number of women	74 (78.7%)	20 (21.3%)	
Maternal age (in year)	24.3 ± 4.4 (17–39)	26.2 ± 6.3 (16–41)	0.124
Parity	1.2 ± 1.3 (0–5)	2.4 ± 1.8 (0–6)	0.0011
Gestational age (week)	9.6 ± 2.0 (5–13)	16.2 ± 2.5 (14–22)	0.0001
Antibiotic coverage	54 women (73%)	17 women (85%)	
Duration of antibiotic coverage (day)	3.2 ± 3.5 (1–12)	4.3 ± 3.8 (1–14)	0.22
Time from abortion to consultation (day)	11.9 ± 13.4 (0–76)	14.5 ± 20.0 (0–90)	-
Hospital stay (day)	3.3 ± 3.0 (0–17)	3.7 ± 4.7 (1–21)	0.64

FTA: First trimester abortions.

STA: Second trimester abortions.

Concerning marital status, six women (30%) were married in the STA group as opposed to 13 (17.6%) in the FTA group.

The main abortion providers were nurses (Table 2). STA was usually conducted in patients’ homes and in primary health centers (Table 3). Main methods used for terminating pregnancy in the second trimester were dilatation and curettage or dilatation and evacuation (D&C/D&E) with ovum forceps and manual vacuum aspiration (MVA) (Table 4).

**Table 2:** Distribution of abortion’s provider

**Provider of abortion**	**FTA**	**STA**
	**N (%)**	**N (%)**
Nurses	43 (58.1)	15 (75)
General practitioner	11 (14.9)	3 (15)
A friend	8 (10.8)	1 (5)
Self-induction	7 (9.5)	1 (5)
Traditional healers	4 (5.4)	0 (0)
Consultant in Obst & Gyn	1 (1.4)	0 (0)
Total	74 (100)	20 (100)

FTA: First trimester abortions.

STA: Second trimester abortions.

**Table 3:** Places where abortions were carried out

**Place of abortion**	**FTA**	**STA**
	**N (%)**	**N (%)**
Primary health centers	23 (31.1)	10 (50)
Abortionist’s or patient’s home	32 (43.2)	7 (35)
Private clinics	17 (23)	3 (15)
Hospital	2 (2.7)	0 (0)
Total	74 (100)	20 (100)

FTA: First trimester abortions.

STA: Second trimester abortions.

**Table 4:** Distribution of abortion’s method used

**Methods used**	**FTA**	**STA**
	**N (%)**	**N (%)**
Dilatation & curettage/evacuation	20 (27.0)	8 (40)
MVA	33 (44.6)	4 (20)
IM injection*	2 (2.7)	2 (10)
Transcervical foreign body**	3 (4.0)	2 (10)
Misoprostol	11 (14.9)	2 (10)
Amniotomy	0 (0)	2 (10)
Traditional methods	4 (5.4)	0 (0)
Potassium permanganate***	1 (1.4)	0 (0)
Total	74 (100)	20 (100)

FTA: First trimester abortions; STA: Second trimester abortions; MVA: Manual vacuum aspiration, IM: Intramuscular.

*Drug used unspecified.

**Various objects used.

***Inserted in the vagina.

Out of the eight cases of STA carried out with D&C/D&E, seven (87.5%) were performed by nurses resulting in two uterine perforations with generalized peritonitis, five incomplete abortions complicated either with severe anemia, hypovolemic shock (systolic blood pressure <80 mm Hg) or pelvic infection. The case of maternal death after D&C was performed by a general practitioner. The four STA performed with MVA were done by nurses and all ended in incomplete abortions and severe anemia. The two cases of STA by amniotomy were carried out by general practitioners and resulted in expulsion of the fetus but with severe anemia in one case and incomplete abortion in the other. The two cases of STA performed with misoprostol (by a nurse in one case and by self induction in the second case) resulted in septic incomplete abortion in one case and mild anemia in the second case. Hypovolemic shock was recorded in the two cases of STA performed by nurses by an intramuscular injection of an unspecified medication. Lastly, the two STA done by transcervical foreign body insertion were performed by a nurse and a friend respectively and ended in incomplete abortions and severe anemia.

The most severe complications observed were maternal anemia and death (Table 5). The two cases of maternal death were due to generalized body swelling (autopsy not done) that followed an intramuscular injection of a non specified medication at 8 weeks gestation in one case and in the other case, to septic shock (Blood pressure fluctuating with systolic blood pressure <80 mmHg associated with pulse rate ≥110/minute and hyperthermia or hypothermia) that developed after generalized peritonitis following curettage at 14 weeks.

**Table 5:** Abortion complications in relation to trimester

**Complications**	**FTA**	**STA**
	**N (%)**	**N (%)**
Maternal death	1 (1.4)	1 (5)
Severe anemia	19 (25.7)	9 (45)
Uterine perforation	4 (5.4)	2 (10)
Hypovolemic shock	11 (14.9)	5 (25)
Incomplete abortion	15 (20.3)	1 (5)
Pelvic infection	16 (21.6)	1 (5)
Generalized peritonitis	2 (2.7)	0 (0)
Septic shock	2 (2.7)	0 (0)
Septic incomplete abortion	1 (1.4)	0 (0)
Mild anemia (Hb: 9 to <11 g/dl)	3 (4.0)	1 (5)
Total	74 (100)	20 (100)

FTA: First trimester abortions.

STA: Second trimester abortions.

The longest admission duration (21 days) was observed in the STA group with the patient who died of a septic shock. In fact after abortion that patient had a left tubo-ovarian abscess that ruptured and gave rise to generalized peritonitis, necessitating a laparotomy which was subsequently complicated by evisceration, after which a second laparotomy was carried out. During this second post operative period, she developed a septic shock and died. The longest admission duration in the FTA group was due to the management of an acute renal failure that followed a septic shock after an aspiration was done at 10 weeks.

## Discussion

In our study 21.3% of women (20) had second trimester abortions (STA). This rate is close to that found in South Africa where 25% of abortions were STA [11], but higher than figures for USA in 2008 which revealed that 10.3% of abortions were STA with only 4% at 16 weeks and above [10]. The limit between the first and the second trimesters varies from 12 to 13 weeks according to authors. In our study, we used a cutoff point of 13 weeks given that it was used by some authors [10]. This high rate of second trimester abortions in our series might be explained by the late recognition of pregnancy, the lack of financial means to quickly perform the abortion, or the delay in discreetly looking for a provider of abortion [7]. In our country, few women use a contraceptive method soon after delivery, despite the fact that most resume with sexual intercourse before the return of their normal cycle. Some of them conceive and realize that they are pregnant only with quickening (16 to 20 weeks). This is contrary to the situation in USA and South Africa, where women frequently use contraception, many second trimester abortions are frequently wanted pregnancies and abortion is not illegal [10,11]. Moreover, because of under employment and poverty in our country, some women, despite early discovery of the pregnancy might delay in obtaining financial means necessary to terminate that pregnancy [7]. Finally, given that abortion is illegal in our context, women might be afraid to consult obstetricians and gynecologists in health facilities where police stations are usually found.

There was no statistically significant difference regarding maternal age between the 2 groups. This is contrary to observations made by Uria in Spain who noticed that late abortions occurred more frequently among teenagers and women of low education [15].

Concerning marital status STA was more observed among married women as also observed in India [16]. This might be due in our series, to the late recognition of pregnancy status (some might have conceived few months after a delivery before the return of their normal cycle) or to the fact that married women might be hesitating for longer periods between pregnancy termination and conservation. This is often observed in our setting among women with four or more living children who sometimes hesitate for long, as concerns having future childbirths. Contrarily in rural India, unsuccessful prior attempts to terminate the pregnancy, poverty, limited access to health facilities and sex selection were significantly associated with second trimester abortion [16]. FTA in our study was usually carried out by single women (82.4%). These women might have taken their decision to terminate the pregnancy, without any hesitation.

The mean gestational age of STA in our study (16.2 weeks) was slightly lower than that found by Simetka et al. in their series (18 ± 1 weeks) [17]. In our series STA were preferentially done by nurses (75%). This could be explained by the fact that they might either be less aware of the possible STA complications, or be more accessible than doctors, given the insufficient number of the latter in Cameroon (particularly the specialists in obstetrics and gynecology) [18]. Finally, nurses are usually cheaper than doctors, hence, they might be more accessible. Very poor women sometimes choose to terminate their pregnancy themselves or with the help of a friend.

Abortions were commonly carried out in patients’ homes (by nurses, by a friend, or by self induction), in primary health centers (where only nurses are found) and in private clinics (by doctors) and rarely in hospitals (by doctors). This is due to the fact that since abortion is illegal in Cameroon, it should be performed where there is maximum confidentiality. The methods more used for STA in our study were an intramuscular injection by nurses of an unspecified medication, transcervical foreign body insertion by nurses, dilatation & and curettage or evacuation by nurses and doctors and amniotomy (by doctors). In the past two decades, misoprostol has been very effective in terminating pregnancy at various gestational ages [19]. Misoprostol is still the drug of choice for terminating pregnancy in the second trimester in other studies [11,19]. In our series, misoprostol was not commonly used in terminating STA. This can be explained by the fact that most abortion providers were nurses (Table 3), who might have had limited knowledge on pregnancy termination using this drug. In Cameroon, nurses are not trained for abortion. Therefore, they should be trained in uterine evacuation procedures in order to reduce abortion related complications, as this has been done elsewhere [20]. Surgical methods used (dilatation and curettage or evacuation and MVA) may be very difficult if done by untrained health providers (nurses) especially in the second trimester. This may explain the so many complications observed. A high number of complications associated with D&C was noticed elsewhere [21]. Duration of antibiotic coverage was usually short in both groups (mean duration <5 days). This could explain the infectious complications found in both groups especially in women with FTA given that the mean time period that elapsed between abortion and consultation in our services was usually long (11.9 days for FTA and 14.5 for STA). An incomplete abortion, especially when carried out without aseptic measures can easily be complicated with endometritis which can evolve to salpingitis, peritonitis and septic shock. Since the abortions were clandestinely carried out, we cannot be certain if asepsis was respected.

Severe complications of STA were hemorrhage complicated with severe anemia or hypovolemic shock, uterine perforation and maternal death (Table 4). Severe anemia and hypovolemic shock resulted from an incomplete abortion. This shows that many abortion providers (mostly nurses than general practitioners) did not know the signs of a emptied uterus. Uterine perforations occurred with dilatation and curettage in six cases (five cases by a nurse and one case by a general practitioner). This is not surprising since in Cameroon, nurses during their training receive almost no knowledge on anatomy of the uterus and on how to empty a uterus with curettage. The two cases of generalized peritonitis followed uterine perforations. This might have been favored by working in septic conditions and short term antibiotic coverage. Hospital stay was slightly prolonged in STA than in FTA.

The long time that elapsed between abortion and consultation in our study might be explained by the fact that since abortion is illegal, women are afraid to be blamed, should they report to the hospital. Moreover, even if they decided to seek for help in our services, they might lack financial means to consult [7]. They therefore stay at home hoping for a spontaneous improvement while carrying out auto medication. It is usually when they found their lives endangered (persistent vaginal bleeding, fever or fatigue) that most of them consulted. This may explain the high rate of severe anemia and hypovolemic shock observed. In our study, one patient consulted in our service 90 days after the abortion was done.

Some limitations that may exist in our study are the fact that since the abortions were clandestinely carried out, some of the information found in the patients’ file might not be accurate enough, for instance the duration of antibiotic coverage, and we have no means to verify their veracity. Also, there is a small sample size especially with respect to the STA.

## Conclusion

STA were commoner among married women and were more usually done in primary health centers using dilatation and curettage or evacuation with forceps, MVA, transcervical foreign body insertion, intramuscular injection of an unspecified medication or amniotomy followed by spontaneous expulsion of the product of conception. Abortions were usually performed by nurses. Complications of STA were: hemorrhage complicated by anemia or hypovolemic shock, uterine perforation and maternal death. Given the high rate of complications associated with clandestine abortions, women should be advised to seek for help from trained health personnel (Gynecologists Obstetricians). Moreover, nurses should be trained in uterine evacuation procedures in order to reduce abortion related complications. Nurses should also be advised to refer women seeking STA to Gynecologists and Obstetricians. Finally, to reduce the prevalence of abortion in general, the government should make contraception available to all women, as well as use public media to sensitize women on the dangers of abortion and on the need of using family planning services.

## Abbreviations

STA: Second trimester abortions; FTA: First trimester abortions; SD: Standard deviation; D&C: Dilatation & Curettage; D&E: Dilatation & evacuation; MVA: Manual vacuum aspiration.

## Competing interests

The authors declare that they have no competing interest.

## Authors’ contributions

EN conceived the study, performed acquisition, analysis and interpretation of data, REM has been involved in drafting the manuscript, JNF revised the manuscript critically. All authors read and approved the final manuscript.

## Pre-publication history

The pre-publication history for this paper can be accessed here:

http://www.biomedcentral.com/1472-6874/14/108/prepub

## Supplementary Material

Additional file 1: STROBE Statement—checklist of items that should be included in reports of observational studies. Click here for file
