# Supplementary material for: How risky are second trimester clandestine abortions in Cameroon: a retrospective descriptive study
Source: BMC Womens Health. 2014 Sep 9;14:108. doi: 10.1186/1472-6874-14-108 (PMC4166018; doi:10.1186/1472-6874-14-108)
Supplement: Additional file 1: — STROBE Statement—checklist of items that should be included in reports of observational studies. [file 1472-6874-14-108-S1.doc]

STROBE Statement—checklist of items that should be included in reports of observational studies

| | **Title and abstract** | (*a*) Indicate the study’s design with a commonly used term in the title or the abstract: Retrospective descriptive study | x | | --- | --- | --- | | (*b*) Provide in the abstract an informative and balanced summary of what was done and what was found. The abstract contains these informations | x | | Background/rationale | Explain the scientific background and rationale for the investigation being reported: Done | x | | Objectives | State specific objectives, including any prespecified hypotheses: Done | x | | Study design | Present key elements of study design early in the paper: Done | x | | Setting | Describe the setting, locations, and relevant dates, including periods of recruitment, exposure, follow-up, and data collection: Done in the methods section | x | | Participants | Women who carried out clandestine abortions | x | | Variables | Clearly define all outcomes, exposures, predictors, potential confounders, and effect modifiers. Give diagnostic criteria, if applicable: Done | x | | Data sources/ measurement | For each variable of interest, give sources of data and details of methods of assessment (measurement). Describe comparability of assessment methods if there is more than one group: Done | x | | Bias | Describe any efforts to address potential sources of bias: Done in the methods section | x | | Study size | Explain how the study size was arrived at: Done | x | | Quantitative variables | Explain how quantitative variables were handled in the analyses. If applicable, describe which groupings were chosen and why | x | | Statistical methods | Describe any methods used to examine subgroups and interactions | x | | Descriptive data | Give characteristics of study participants (eg demographic, clinical, social) and information on exposures and potential confounders: Done | x | | Outcome data | Report numbers of outcome events or summary measures: Done | x | | Main results | Description | x | | Discussion Key results | Summarise key results with reference to study objectives: Done | x | | Limitations | Discuss limitations of the study, taking into account sources of potential bias or imprecision. Discuss both direction and magnitude of any potential bias: Done | x | | Interpretation | Give a cautious overall interpretation of results considering objectives, limitations, multiplicity of analyses, results from similar studies, and other relevant evidence: Done | x | | Generalisability | Discuss the generalisability (external validity) of the study results | NA | | Funding | Give the source of funding and the role of the funders for the present study and, if applicable, for the original study on which the present article is based: No conflicts of interest to declare. | x | |  |  |
| --- | --- | --- | --- | --- | --- | --- | --- | --- | --- | --- | --- | --- | --- | --- | --- | --- | --- | --- | --- | --- | --- | --- | --- | --- | --- | --- | --- | --- | --- | --- | --- | --- | --- | --- | --- | --- | --- | --- | --- | --- | --- | --- | --- | --- | --- | --- | --- | --- | --- | --- | --- | --- | --- | --- | --- | --- | --- | --- | --- | --- | --- | --- | --- | --- |
